# Survival outcomes of patients with head and neck squamous cell cancer with hepatitis B virus infection: An analysis from an endemic tertiary center

**DOI:** 10.1002/cam4.5469

**Published:** 2022-11-24

**Authors:** Cheng‐Lun Lai, Cheng‐Hsien Lin, Yu‐Chen Su, Yu‐Hsuan Shih, Chen‐Chi Wang, Chieh‐Lin Jerry Teng, Cheng‐Wei Chou

**Affiliations:** ^1^ Division of Hematology/Medical Oncology, Department of Medicine Taichung Veterans General Hospital Taichung Taiwan; ^2^ Department of Post‐Baccalaureate Medicine, College of Medicine National Chung Hsing University Taipei Taiwan; ^3^ School of Medicine National Yang Ming Chiao Tung University Taichung Taiwan; ^4^ School of Speech Language Pathology & Audiology Chung Shan Medical University Taichung Taiwan; ^5^ Department of Audiology and Speech‐Language Pathology Asia University Taichung Taiwan; ^6^ Department of Otolaryngology‐Head & Neck Surgery Taichung Veterans General Hospital Taichung Taiwan; ^7^ Department of Life Science Tunghai University Taichung Taiwan; ^8^ School of Medicine Chung Shan Medical University Taichung Taiwan; ^9^ Graduate Institute of Biomedical Sciences China Medical University Taichung Taiwan

**Keywords:** cirrhosis, head and neck squamous cell cancer (HNSCC), hepatic dysfunction, hepatitis B virus (HBV), immune checkpoint inhibitor (ICI), survival

## Abstract

**Background:**

Hepatitis B virus (HBV) affects the occurrence and survival outcome of various malignant disorders. The study aimed to evaluate the survival outcome of head and neck squamous cell cancer (HNSCC) patients with or without HBV infection.

**Methods:**

This study included patients with HNSCC who visited Taichung Veterans General Hospital from 2007 to 2015. HBV infection was defined by hepatitis B surface antigen (HBsAg) seropositivity. By propensity score matching, we compared survival outcomes, including progression‐free survival (PFS) and overall survival (OS), among patients with or without HBV infection.

**Results:**

The prevalence of HBV infection in our cohort was 12.3%. Among the 1,015 patients included in the matched analysis, a higher risk of baseline liver cirrhosis (11.3% vs. 3.4%, *p* < 0.001) and initial hepatic dysfunction (10.8% vs. 5.4%, *p* = 0.005) rates were observed than those without HBV infection at baseline. The 5‐year OS was 43.1% and 53.2% (*p* < 0.001) and the 5‐year PFS was 37.4% and 42.3% (*p* = 0.007) in patients with and without HBV infection, respectively. The incidence of subsequent hepatic dysfunction showed no difference between patients with and without HBV infection (29.6% vs. 26.8%, *p* = 0.439).

**Conclusions:**

Patients with HNSCC and HBV infection were younger and had a higher risk of cirrhosis compared to those without HBV infection. Moreover, HBV infection significantly influenced the OS and PFS outcomes but not subsequent hepatic dysfunction in patients with HNSCC.

## Graphical abstract

5‐year overall survival in hepatitis B surface antigen (HBsAg)‐positive and HBsAg‐negative patients with head and neck squamous cell carcinoma (HNSCC).

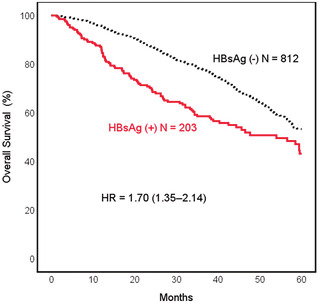

## 1 INTRODUCTION

Head and neck squamous cell carcinoma (HNSCC) is the sixth leading cause of malignancy, with a male predominance of approximately 90%.^1^ The risk factors for HNSCC include alcohol, tobacco product, betel nut consumption, and infection with oncoviruses such as the Epstein–Barr virus, human papillomavirus (HPV), and hepatitis C virus (HCV).^2^

Chronic hepatitis B virus (HBV) infection is a crucial health concern, especially in Asia. HBV infection correlates significantly with chronic hepatitis, cirrhosis, and hepatocellular carcinoma (HCC),^3^ particularly in endemic areas like Taiwan. Although Taiwan was the first country to initiate a nationwide vaccination project against HBV in July 1984,^4^ the seropositivity rate of hepatitis B surface antigen (HBsAg) in Taiwan remains between 13.7 and 17.3%.^5^, ^6^

HBV infection is also associated with several malignancies, particularly in endemic areas as compared to the non‐endemic areas. HBV infection is carcinogenic and associated with an increased risk of HCC, lymphoma, oral cancer, and gastrointestinal cancers including stomach cancer, colorectal cancer, and pancreatic cancer.^7^ HBV infection correlates with a poor prognostic outcome in patients with pancreatic cancer,^8^ nasopharyngeal carcinoma (NPC),^9^ advanced non‐small cell lung cancer (NSCLC),^10^ breast cancer,^11^ and ovarian cancer.^12^ In contrast, HBV infection is reportedly an independent favorable prognostic factor in patients with esophageal cancer who underwent esophagectomy,^13^ with no survival influence in patients with squamous cell cervical cancer.^14^

Nevertheless, only a few clinical studies have investigated the outcomes of patients with HNSCC and HBV infection. The impact of HBV infection on the outcome of HNSCC remains unknown. Therefore, we conducted this retrospective study to determine the association of HBV infection with clinical characteristics, survival outcomes, and hepatic function in patients with HNSCC.

## 2 MATERIALS AND METHODS

### 2.1 Patient selection

**FIGURE 1:**
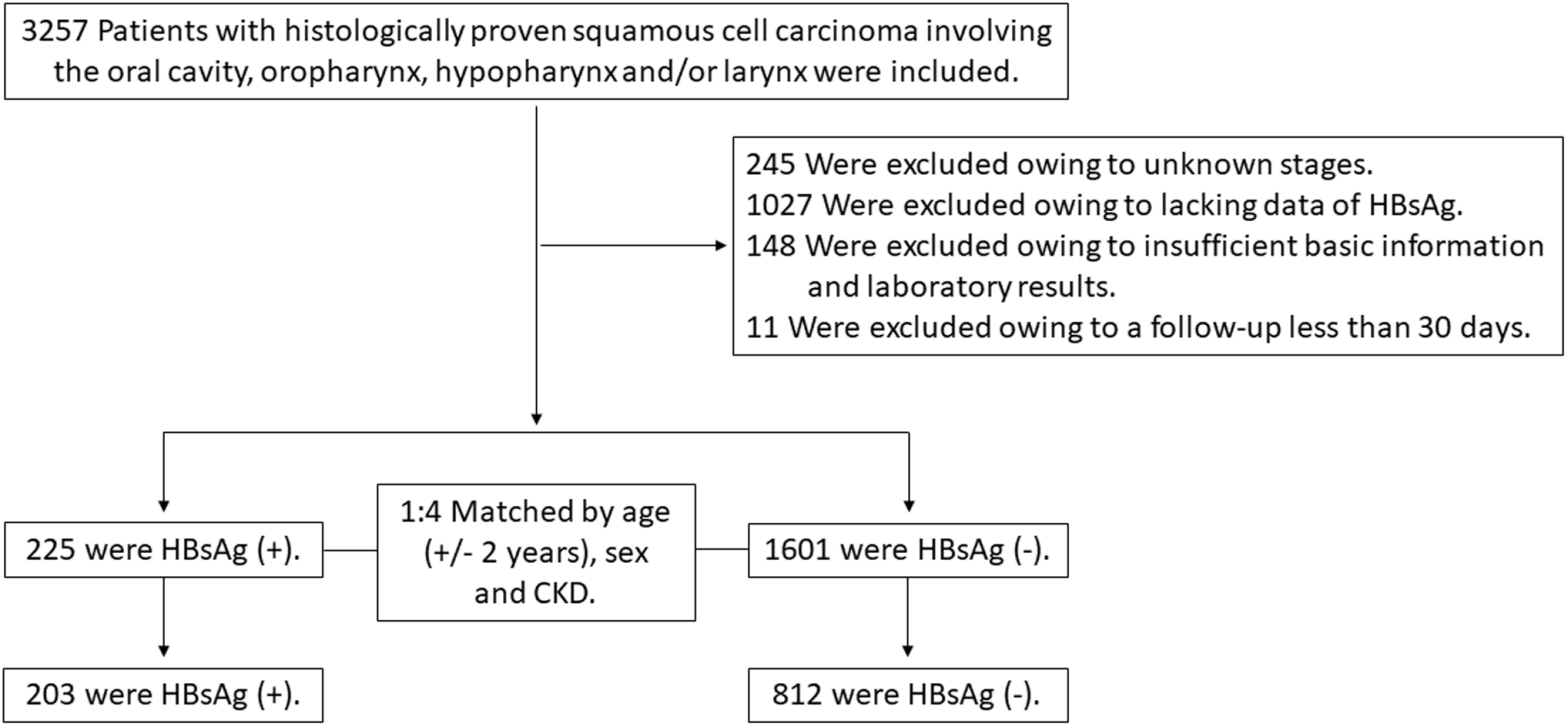
Study flow diagram.

We conducted a retrospective study using data from the cancer registry database and medical records at Taichung Veterans General Hospital from 2007 to 2015 with follow‐up for 5 years till December 31, 2020. Patients with human immunodeficiency virus infection or known cancers other than HNSCC were excluded. A total of 1985 patients with histologically confirmed squamous cell carcinoma involving the oral cavity, oropharynx, hypopharynx, and/or larynx were included. Of them, we excluded 159 patients due to insufficient data on basic information and clinical laboratory test results or a follow‐up duration of fewer than 30 days. We included the remaining 1826 patients by the propensity score matched method using a 1:4 ratio matched by age, sex, and comorbidity (Figure 1). A total of 203 HBsAg‐positive patients and 812 HBsAg‐negative patients were analyzed. The patient's cancer stage was determined on the basis of the American Joint Committee on Cancer TNM staging classification (eighth edition). Moreover, treatment strategies and comorbidities including cardiovascular diseases, chronic obstructive pulmonary disease, chronic kidney disease, hypertension, and diabetes mellitus were also recorded.

The review board of Taichung Veterans General Hospital approved the retrospective study design, and the requirement for informed consent was waived by the institutional review board.

### 2.2 Virologic and liver function studies

All patients were tested for HBsAg and hepatitis C virus antibody (anti‐HCV) by the electrochemiluminescence immunoassay during diagnosis. We tested the viral loads of HBV and HCV using real‐time polymerase chain reaction analysis, although this was not performed in all patients. Based on the American Association for the Study of Liver Diseases hepatitis B guidelines (2018),^15^ we defined chronic HBV infection as HBsAg seropositivity and active HBV carrier as HBsAg seropositivity combined with a HBV viral load >2000 IU/ml. The definition of HBV reactivation was (a) a 100‐fold increase in HBV viral load than the baseline level, (b) a HBV viral load ≥1000 IU/ml with previously undetectable levels, or (c) a HBV viral load ≥10,000 IU/ml if the baseline level was unavailable.

All the patients underwent liver and renal function tests at diagnosis as part of the clinical routine care. Cirrhosis was defined based on typical radiologic findings, including hepatic nodularity, massive ascites, and splenomegaly on abdominal or liver ultrasonography, computed tomography, or magnetic resonance imaging. Hepatic dysfunction, hypoalbuminemia, and renal function impairment were defined as grade 1 or above adverse events according to the common terminology criteria for adverse events version 5.0.^16^

### 2.3 Survival and statistical analysis

The follow‐up duration was based on the date of HNSCC diagnosis to either the date of death or the date of the last follow‐up on December 31, 2020. To evaluate the influence of HBsAg seropositivity on the survival outcomes of patients with HNSCC, we assessed the progression‐free survival (PFS) and overall survival (OS) as primary endpoints. The PFS was calculated from the day of cancer diagnosis to the date of disease progression, relapse, or death, whichever occurred first. The OS was calculated from the date of cancer diagnosis to the date of death. We also assessed the liver functions as secondary endpoints.

We used SPSS Statistics for Windows, version 26.0. (IBM, Armonk, NY) for all statistical analyses. We used the chi‐square test for comparing categorical variables and the independent sample *t*‐test for comparing continuous variables between the groups. The Kaplan–Meier method was used to calculate the actuarial rates, and differences between groups were compared by the log‐rank test. Multivariate analysis using the Cox proportional‐hazards model was used to determine the independent significance of variables for OS (age, sex, stage, operation, liver function, renal function, and hepatitis). We determined risk factors for hepatic dysfunction in the HBsAg‐positive group using logistic regression analysis. We set the significance level as *p <* 0.05.

## 3 RESULTS

### 3.1 Patient characteristics and comparison between the HBsAg‐positive and HBsAg‐negative groups

The demographic, oncologic, and hepatic characteristics of all 1,826 patients are listed in Tables S1 and S2. The median age at diagnosis of HNSCC was 53.9 years in all patients. Most patients were men; the most common primary cancer site was that of the oral cavity, and approximately half of the patients were first diagnosed with stage IV disease. A total of 225 of 1,826 patients (12.3%) showed HBsAg seropositivity. The median follow‐up time was 31.9 months (interquartile range [IQR], 14.0–46.2 months). During the follow‐up period, 1,118 (61.2%), 128 (56.9%), and 990 (61.8%) patients died during follow‐up in all patients, HBsAg‐positive group, and HBsAg‐negative group, respectively. The HBsAg‐positive group had a higher proportion of patients aged <65 years (88.9% vs. 83.0%, *p* = 0.025). The median ages at diagnosis in the HBsAg‐positive and HBsAg‐negative groups were 51.3 ± 10.7 years and 54.2 ± 11.0 years, respectively (*p* < 0.001). In the matched cohort, the HBsAg‐positive group (n = 203) showed a higher risk of baseline liver cirrhosis (11.3% vs. 3.4%, *p* < 0.001) and initial hepatic dysfunction (10.8% vs. 5.4%, *p* = 0.005) as well as higher alanine transaminase, aspartate transaminase, and international normalized ratio levels than the HBsAg‐negative group (*n* = 812). The other characteristics were similar in both groups (Tables 1 and 2). The risk of subsequent hepatic dysfunction was also similar between HBsAg‐positive and HBsAg‐negative groups in the matched cohort (29.6% vs. 26.8%, *p* = 0.439).

**TABLE 1:** Baseline characteristics of the HBsAg (+) and HBsAg (−) patients with HNSCC

	HBsAg (+) (*n* = 203)	HBsAg (−) (*n* = 812)	*p* ^a^
Age at diagnosis ≥ 65 years, *n* (%)	16 (7.9)	64 (7.9)	1.000
Sex, *n* (%)			1.000
Female	6 (3.0)	24(3.0)	
Male	197 (97.0)	788 (97.0)	
Primary sites, *n* (%)			0.160
Oral cavity	127 (62.6)	543 (66.9)	
Oropharynx	34 (16.7)	132 (16.3)	
Hypopharynx	28 (13.8)	109 (13.4)	
Larynx	14 (6.9)	28 (3.4)	
Differentiation, *n* (%)			0.788
Well	15 (7.4)	54 (6.7)	
Moderate	121 (59.6)	459 (56.5)	
Poor	56 (27.6)	250 (30.8)	
Unknown	11 (5.4)	49 (6.0)	
Tumor stage^b^, *n* (%)			0.829
0/I	47 (23.2)	187 (23.0)	
II	27 (13.3)	115 (14.2)	
III	32 (15.8)	109 (13.4)	
IV	97 (47.8)	401 (49.4)	
Treatment, *n* (%)			
Excisional surgery	146 (71.9)	623 (76.7)	0.153
Systemic therapy^c^	105 (51.7)	446 (54.9)	0.413
Radiation therapy	101 (49.8)	453 (55.8)	0.122
Comorbidity			
CAD	16 (7.9)	77 (9.5)	0.479
COPD	27 (13.3)	120 (14.8)	0.593
CHF	4 (2.0)	18 (2.2)	0.829
CVD	12 (5.9)	58 (7.1)	0.536
CKD	39 (19.2)	156 (19.2)	1.000
HTN	63 (31.0)	268 (33.0)	0.592
DM	42 (20.7)	180 (22.2)	0.649

Abbreviations: −, negative; +, positive; CAD, coronary artery disease; CHF, congestive heart failure; CKD, chronic kidney disease; COPD, chronic obstructive pulmonary disease; CVD, cardiovascular disease; DM, diabetes mellitus; HBsAg, hepatitis B surface antigen; HNSCC, head and neck squamous cell carcinoma; HTN, hypertension.

^a^ *p* values were calculated by using the chi‐square test.

^b^ Tumor stage was assessed based on the 8th Edition of the American Joint Committee on Cancer staging system.

^c^ Systemic therapy included chemotherapies, cetuximab (an anti‐epidermal growth factor receptor antibody), and immune checkpoint inhibitors.

**TABLE 2:** Hepatic and virologic characteristics of the HBsAg (+) and HBsAg (−) patients with HNSCC

	HBsAg (+) (*n* = 203)	HBsAg (−) (*n* = 812)	*p*
Cirrhosis^a^, *n* (%)	23 (11.3)	28 (3.4)	<0.001^b^
Anti‐HCV (+), *n* (%)	13 (6.4)	60 (7.4)	0.627^b^
ALT (U/L)	47.8 ± 64.4	34.5 ± 35.9	0.005^c^
AST (U/L)	38.8 ± 40.3	32.1 ± 37.8	0.025^c^
Total Bil. (mg/dl)	0.7 ± 1.3	0.6 ± 0.5	0.216^c^
ALKP (U/L)	110.3 ± 51.6	110.6 ± 53.8	0.956^c^
Alb (g/dl)	4.0 ± 0.7	4.1 ± 0.7	0.534^c^
INR	1.04 ± 0.2	1.00 ± 0.1	0.005^c^
eGFR (mL/min/1.73 m^2^)^d^	84.8 ± 25.3	82.9 ± 25.3	0.332^c^
Initial hepatic dysfunction^e^, *n* (%)	22 (10.8)	44 (5.4)	0.005^b^
HBV viral load detectable, *n* (%)	85 (41.9)		
HBV active carrier^f^, *n* (%)	40 (19.7)		
HBV treatment received, *n* (%)	108 (53.2)		
Entecavir	91 (44.8)		
Telbivudine	17 (8.4)		
Subsequent hepatic dysfunction, *n* (%)	60 (29.6)	218 (26.8)	0.439^b^
HBV reactivation^g^	13 (6.4)		

*Note*: Numerical data are shown as mean ± standard error of mean.

Abbreviations: −, negative; +, positive; Alb, albumin; ALKP, alkaline phosphatase; ALT, alanine aminotransferase; AST, aspartate aminotransferase; Cr, creatinine; eGFR, estimated glomerular filtration rate; HBsAg, hepatitis B surface antigen; HBV, hepatitis B virus; HCV, hepatitis C virus; HNSCC, head and neck squamous cell carcinoma; INR, international normalized ratio; Total Bil., total bilirubin.

^a^ Cirrhosis was defined based on typical radiologic findings (e.g., hepatic nodularity, massive ascites, and splenomegaly).

^b^ *p* values were calculated by using the chi‐square test.

^c^ *p* values were calculated by using the independent sample *t*‐test.

^d^ eGFR was measured with the modification of diet in renal disease equation.

^e^ Hepatic dysfunction was defined as grade 1 or above of hepatic toxicity based on the common terminology criteria for adverse events versions 5.0.

^f^ HBV active carrier was defined as HBV DNA level > 2000 IU/ml.

^g^ HBV reactivation was diagnosed as (1) a 100‐fold increase in viral load compared to the baseline level, (2) DNA level ≥ 1000 IU/mL with undetectable level at baseline, or (3) DNA level ≥ 10,000 IU/mL if the baseline level was unavailable.

### 3.2 Prognostic impact of chronic HBV infection in patients with HNSCC

**FIGURE 2:**
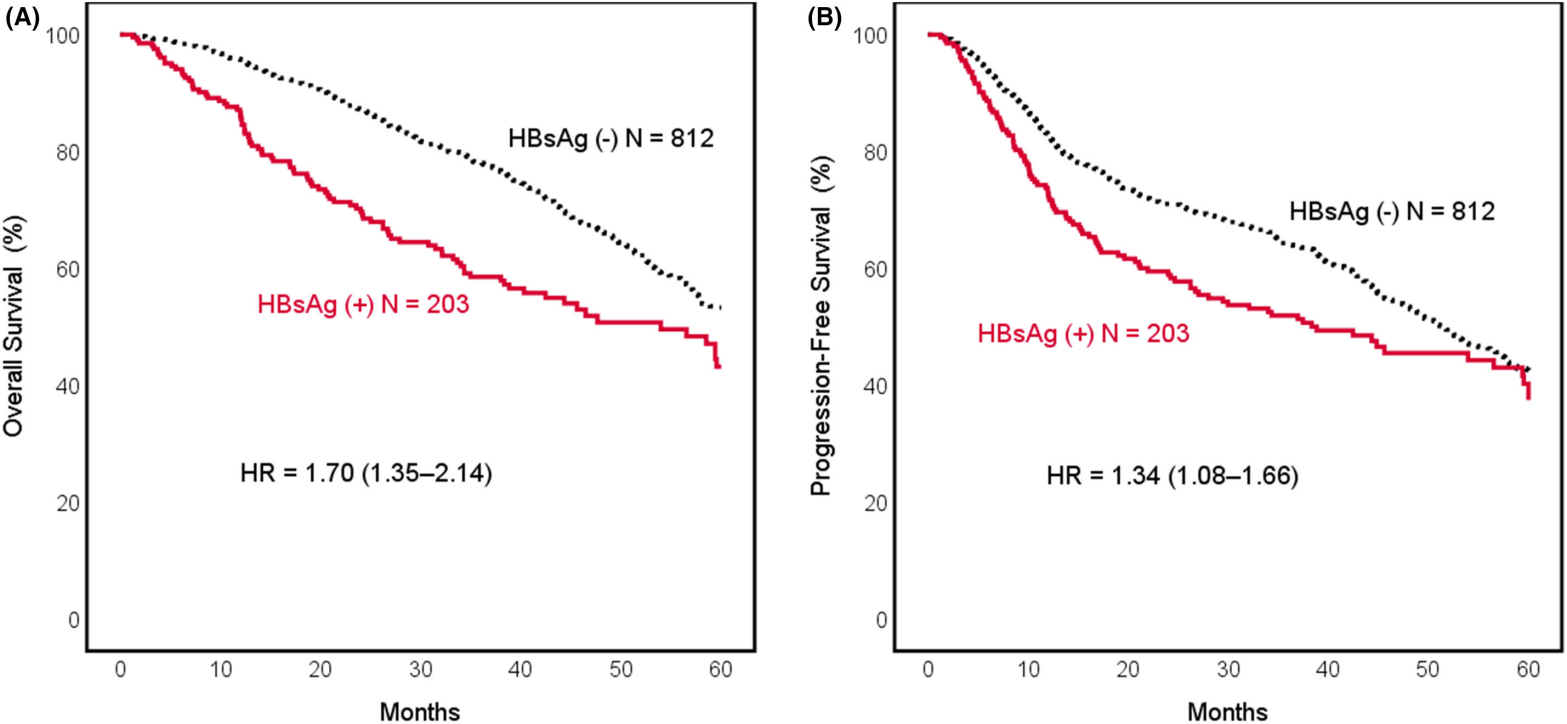
Kaplan–Meier survival curves for (A) 5‐year overall survival (OS) and (B) 5‐year progression‐free survival (PFS) in hepatitis B surface antigen (HBsAg)‐positive and HBsAg‐negative patients with head and neck squamous cell carcinoma (HNSCC). Hazard ratios (HRs) and 95% confidence intervals (CIs) have been calculated by using the Cox regression model.

In the present study, we demonstrated the poor survival impact of HBV infection on patients with HNSCC. The 5‐year OS rate was 43.1% and 53.2% (hazards ratio [HR] = 1.70; 95% confidence interval [CI] = 1.35–2.14; *p* < 0.001, Figure 2A) and the 5‐year PFS was 37.4% and 42.3% (HR = 1.34; 95% CI = 1.08–1.66; *p =* 0.007, Figure 2B) in the HBsAg‐positive and HBsAg‐negative groups, respectively. The median OS was 54.0 months (95% CI = 40.4–65.6) and not reached at 5 years follow‐up; PFS was 38.3 months (95% CI = 21.0–55.7) and 51.4 months (95% CI = 46.9–55.9) in the HBsAg‐positive and HBsAg‐negative groups, respectively.

### 3.3 Prognostic factors for patients with HNSCC

We analyzed clinical variables to identify prognostic factors of 5‐year OS and PFS. We found that HBsAg‐infection and advanced stages were independent adverse prognostic factors for OS and PFS (Tables 3 and 4). However, initial hepatic dysfunction, initial hypoalbuminemia, subsequent hepatic function, cirrhosis, and HCV infection were not significant prognostic factors for poor 5‐year OS and PFS. Therefore, we demonstrated that HBV infection was a factor for poor prognosis in our HNSCC patient cohort.

**TABLE 3:** Poor prognostic factors for 5‐year overall survival

Clinical variables	Univariate analysis			Multivariate analysis		
	HR	95% CI	*p* ^a^	HR	95% CI	*p* ^a^
Age ≥ 65 versus <65 years	1.11	0.79–1.58	0.544			
Sex male versus female	1.02	0.58–1.81	0.943			
Tumor stage^b^						
I/0	1.00					
II	1.13	0.75–1.70	0.564			
III	1.69	1.16–2.47	0.006	1.52	1.04–2.22	0.031
IV	2.63	1.96–3.53	<0.001	2.12	1.56–2.88	<0.001
Excisional surgery	0.41	0.33–0.50	<0.001	0.50	0.41–0.62	<0.001
Initial hepatic dysfunction^c^	1.18	0.81–1.72	0.387			
Initial hypoalbuminemia^c^	1.08	0.77–1.53	0.655			
Subsequent hepatic dysfunction^c^	1.18	0.95–1.45	0.135			
Initial cirrhosis^d^	0.97	0.62–1.52	0.900			
Initial renal impairment^e^	1.05	0.79–1.39	0.732			
HBsAg (+)	1.70	1.35–2.14	<0.001	1.67	1.32–2.11	<0.001
Anti‐HCV (+)	1.02	0.71–1.47	0.928			

Abbreviations: +, positive; CI, confidence interval; eGFR, estimated glomerular filtration rate; HBsAg, hepatitis B surface antigen; HCV, hepatitis C virus; HR, hazard ratio.

^a^ Data were analyzed using the Cox proportional‐hazard regression model.

^b^ Tumor stage was assessed based on the 8th Edition of the American Joint Committee on Cancer staging system.

^c^ Hepatic dysfunction and hypoalbuminemia were defined as grade 1 or above of toxicity based on the Common Terminology Criteria for Adverse Events versions 5.0.

^d^ Cirrhosis was defined based on typical radiologic findings (e.g., hepatic nodularity, massive ascites, and splenomegaly).

^e^ Renal impairment was defined as an eGFR < 60 ml/min/1.73 m^2^, calculated with the modification of diet in renal disease equation.

**TABLE 4:** Poor prognostic factors for 5‐year progression‐free survival

Clinical variables	Univariate analysis			Multivariate analysis		
	HR	95% CI	*p* ^a^	HR	95% CI	*p* ^a^
Age ≥ 65 versus <65 years	0.96	0.70–1.33	0.819			
Sex male versus female	1.17	0.71–1.92	0.541			
Tumor stage^b^						
I/0	1.00					
II	0.89	0.65–1.22	0.473			
III	1.09	0.81–1.48	0.563			
IV	1.37	1.09–1.72	0.007	1.20	0.95–1.52	0.136
Excisional surgery	0.60	0.50–0.72	<0.001	0.64	0.52–0.78	<0.001
Initial hepatic dysfunction^c^	0.99	0.78–1.25	0.925			
Initial hypoalbuminemia^c^	1.15	0.84–1.58	0.370			
Subsequent hepatic dysfunction^c^	1.05	0.75–1.48	0.774			
Initial cirrhosis^d^	0.76	0.49–1.16	0.203			
Initial renal impairment^e^	1.02	0.80–1.32	0.841			
HBsAg (+)	1.34	1.08–1.66	0.007	1.33	1.07–1.64	0.01
Anti‐HCV (+)	1.13	0.83–1.55	0.436			

Abbreviations: +, positive; CI, confidence interval; eGFR, estimated glomerular filtration rate; HBsAg, hepatitis B surface antigen; HCV, hepatitis C virus; HR, hazard ratio.

^a^ Data were analyzed using the Cox proportional‐hazard regression model.

^b^ Tumor stage was assessed based on the 8th Edition of American Joint Committee on Cancer staging system.

^c^ Hepatic dysfunction, hypoalbuminemia and renal impairment were defined as grade 1 or above of toxicity based on the Common Terminology Criteria for Adverse Events (CTCAE) versions 5.0.

^d^ Cirrhosis was defined on the basis of combination of typical radiologic findings (e.g., hepatic nodularity, massive ascites, and splenomegaly).

^e^ Renal impairment was defined as an eGFR <60 ml/min/1.73 m^2^, calculated with the modification of diet in renal disease equation.

### 3.4 Hepatic dysfunction and HBV reactivation in the HBsAg‐positive group

The development of subsequent hepatic dysfunction is an obstacle to further treatment and results in a significant risk for morbidity and mortality. To decipher the underlying factors, patients with initial elevation of liver function, co‐infection of HCV, liver cirrhosis, and administration of systemic chemotherapy were found to show a higher risk of subsequent hepatic dysfunction (Table 5). In contrast, the HBV active carrier status was not a risk factor for subsequent hepatic dysfunction.

**TABLE 5:** Risk factors for hepatic dysfunction in HBsAg (+) HNSCC patient

Variables	OR	95% CI	*p* ^a^
Initial ALT >120 (U/L)^b^	0.43	0.15‐1.24	0.119
Initial AST > 120 (U/L)^b^	3.65	1.41‐9.50	0.008
Initial Total Bil. > 1.8 (mg/dl)^b^	1.44	0.55‐3.79	0.455
Initial INR >1.5^b^	3.22	0.50–20.9	0.222
Initial Alb < 3.0 (mg/dl)^b^	1.59	1.00‐2.55	0.051
HBV active carrier^c^	1.29	0.63–2.63	0.483
Anti‐HCV (+)	2.08	1.24–3.50	0.005
Cirrhosis^d^	4.43	2.36‐8.32	<0.001
Initial renal impairment^e^	1.00	0.65–1.54	0.988
Systemic therapy received	1.60	1.19–2.15	0.002

Abbreviations: +, positive; ALT, alanine aminotransferase; AST, aspartate aminotransferase; CI, confidence interval; HBsAg, hepatitis B surface antigen; HBsAg, hepatitis B surface antigen; HBV, hepatitis B virus; HCV, hepatitis C virus; HNSCC, head and neck squamous cell carcinoma; INR, international normalized ratio; OR, odds ratio; Total Bil., total bilirubin.

^a^ *p* values were calculated by logistic regression.

^b^ Cutoff‐point of variables were defined as grade 1 or above of toxicity based on Common Terminology Criteria for Adverse Events versions 5.0.

^c^ HBV active carrier was defined as HBV DNA > 2000 IU/ml.

^d^ Cirrhosis was diagnosed on the basis of combination of typical radiologic findings (e.g., hepatic nodularity, massive ascites, and splenomegaly).

^e^ Renal impairment was diagnosed as eGFR < 60 ml/min/1.73 m^2^, calculated with the modification of diet in renal disease equation.

We observed HBV reactivation in 13 (6.4%) patients with HBsAg‐positive HNSCC. No HBV reactivation was observed in the HBsAg‐negative group, even in those with occult HBV infection (hepatitis B core antibody seropositive patients).

## 4 DISCUSSION

In the present study, HBV infection was an independent unfavorable factor for survival outcomes in patients with HNSCC. Cirrhosis and hepatic dysfunction were more common in the HNSCC patients with HBV infection than those without HBV infection but these factors had no significant influence on survival.

The actual mechanisms underlying the prognostic value of HBV infection in patients with cancer remain unclear. Possible mechanisms include the upregulation of programmed cell death protein 1 and the under‐production of interleukin‐12 due to immunologic dysfunction in patients with HBV infection.^17^, ^18^ Furthermore, HBV reactivation after anti‐cancer therapy might cause subsequent deterioration of liver function and liver cirrhosis.^19^ Due to limited research on the impact of HBV infection on survival and hepatic dysfunction in patients with HNSCC, we retrospectively studied HNSCC patients in our institute. We demonstrated that chronic HBV infection was a predictive factor for increased mortality and cancer progression in patients with HNSCC. Furthermore, patients with HBsAg‐positive HNSCC were younger and more commonly showed initial liver cirrhosis and hepatic dysfunction than patients with HBsAg‐negative HNSCC. However, the frequency of development of subsequent hepatic dysfunction was similar between the two groups. Generally, we observed a higher risk of subsequent hepatic dysfunction in patients with advanced disease who required systemic therapy.

Before the initiation of the national HBV vaccination program, the seropositivity rate of HBV in Taiwan was 17.3%.^6^ The patients in our cohort were all born before July 1, 1984, and had not received HBV vaccination. The seropositivity rate of HBsAg was 12.3% in our cohort, similar to the general population. In two case–control studies conducted in Japan and Korea, there were no significant correlations between HBV infection and HNSCC diagnosis.^20^, ^21^ However, HBV is a carcinogenic virus and a risk factor for several malignant disorders. We found no significant correlation between HBV infection and HNSCC development in the present study.

In a large‐scale study, patients with HBsAg seropositivity showed a lower median age at diagnosis than those without it among all cancer types.^22^ The same result was also obtained in patients with nasopharyngeal cancer.^9^ Our study revealed a significantly younger age at diagnosis in patients with HBsAg‐positive HNSCC than in patients with HBsAg‐negative HNSCC, compatible with the results of other studies. The impact of HBV infection on carcinogenesis has been well‐studied for HCC. Virus DNA and proteins were found in the extrahepatic tissues, including the hematopoietic system, kidney, skin, gastrointestinal tract, and testes, of the HBV‐infected patients.^23^ The detailed underlying mechanisms of the correlation between HBV infection and the carcinogenesis of malignancies require further investigation.

CheckMate 141 and KEYNOTE‐048 are two landmark studies on immune checkpoint inhibitor (ICI) treatment for HNSCC, and ICIs have become the standard treatment choices in metastatic or recurrent HNSCC.^24^, ^25^ However, patients with HBsAg seropositivity were not enrolled in both of these studies; thus, the efficacy and safety of ICIs in the patients are unknown. In our study, only 38 (2.1%) patients received ICIs because they were not reimbursed. Four patients were HBsAg seropositive; they received antiviral treatment and none of them developed HBV reactivation. Previous studies have also revealed that regular viral load monitoring and antiviral prophylaxis enable the safe use of ICIs in patients with HBsAg‐positive cancer.^26^, ^27^ The difference in efficacy and adverse events of ICIs between patients with HBsAg‐positive and HBsAg‐negative HNSCC may be a future research topic.

The anti‐HCV seropositivity rate was 7.3% in our cohort and 4.4% of the general population in Taiwan.^6^ In several retrospective studies, patients with HNSCC had a higher prevalence of HCV infection than the control population without cancer.^28^, ^29^, ^30^, ^31^ HCV infection had no impact on the prognosis of HNSCC patients with different tumor origin sites.^29^, ^30^ However, in patients with oropharyngeal cancers, HCV infection correlated with an increased risk of cancer‐specific death and disease progression.^32^ However, the oncologic significance of this is unclear. In our study, anti‐HCV seropositivity had no unfavorable impact on the 5‐year OS and PFS, even in the oropharyngeal subgroup.

Cirrhosis and hepatic dysfunction, regardless of whether they were initially or subsequently identified, were not poor prognostic risk factors for OS or PFS in our study. However, liver cirrhosis and its severity can predict the risk of mortality in patients with oral cancer receiving chemotherapy.^33^ Previous studies show that the more advanced the cirrhosis and HNSCC, the higher the complication rate after tumor resection.^34^, ^35^, ^36^ In our cohort, cirrhosis was a risk factor for subsequent hepatic dysfunction among patients with HBsAg‐positive HNSCC despite therapy. We demonstrated a similar incidence of HBV reactivation to a previous report in an endemic area of the nasopharyngeal carcinoma cohort (9.1%) in China.^37^ Altogether, patients with adverse risk factors and comorbidities need to be closely monitored during the treatment course.

This study had some limitations. First, it was a retrospective study from an endemic tertiary hospital, and selection bias should therefore be considered. Second, we did not analyze the effects of HPV infection and cigarette and alcohol consumption because these data were lacking for most of the patients. Third, because our data were retrospectively retrieved from a cancer registry database, we could not evaluate the sequence of radiotherapy and systemic therapy. Therefore, survival analysis according to radiotherapy and systemic therapy was not performed. Fourth, antiviral medications were prescribed to a limited number of patients, and the timing of their administration was unknown. Hence, the influence of antiviral prophylaxis or treatment on survival remains unknown. Fifth, hepatic dysfunction might be underestimated because it was defined based solely on laboratory examination findings, not hepatic encephalopathy or the amount of ascites.

## 5 CONCLUSION

In our study, we showed that HBV infection was an independently unfavorable factor for survival outcomes in patients with HNSCC. Cirrhosis and hepatic dysfunction were more common in patients with HNSCC who had HBV infection than those who did not have HBV infection but it had no significant influence on survival. The detailed underlying mechanism for HBV infection and cancer outcomes might need further investigation.

## AUTHOR CONTRIBUTIONS

**Cheng‐Lun Lai**: data curation, data analysis, visualization, and draft writing. **Cheng‐Hsien Lin**: project administration and review. **Yu‐Chen Su**: data analysis and visualization. **Yu‐Hsuan Shih**: project administration and supervision. **Chen‐Chi Wang**: revision of the article for important content. **Chieh‐Lin Jerry Teng**: conceptualization and supervision. **Cheng‐Wei Chou**: conceptualization, project administration, review, and manuscript editing.

## FUNDING STATEMENT

This research received support by Taichung Veterans General Hospital [grant number TCVGH‐1113702B and TCVGH‐NHRI111007 to C.W.C].

## CONFLICT OF INTEREST

The authors disclose no conflicts of interest.

## ETHICS APPROVAL STATEMENT

The institutional review board of Taichung Veterans General Hospital approved this study.

## PATIENT CONSENT STATEMENT

The patient's informed consent was waived by the review board of Taichung Veterans General Hospital.

## Supporting information


Table S1.
Table S2.

## Data Availability

The data in this study cannot be shared because of the privacy and ethical regulation.
