# Supplementary material for: Survival outcomes of patients with head and neck squamous cell cancer with hepatitis B virus infection: An analysis from an endemic tertiary center
Source: Cancer Med. 2022 Nov 24;12(6):6802–10. doi: 10.1002/cam4.5469 (PMC10067106; doi:10.1002/cam4.5469)
Supplement: Supplementary file 1 — Table S1. Table S2. [file CAM4-12-6802-s001.docx]

| **Table 1. Baseline characteristics of the HBsAg (+) and HBsAg (-) patients with HNSCC** | | | | |
| --- | --- | --- | --- | --- |
|  | **HBsAg (+)**  **(n = 225)** | **HBsAg (-)**  **(n = 1601)** | | ***p****^a^* |
| Age at **diagnosis ≥ 65 years, n (%)** | 25 (11.1) | 272 (17.0) | | 0.025 |
| **Sex, n (%)** | | | 0.169 | |
| Female | 19 (8.4) | 97 (6.1) | |  |
| Male | 206 (91.6) | 1504 (93.9) | |  |
| **Primary sites, n (%)** | | | 0.845 | |
| Oral cavity | 143 (63.6) | 1004 (62.7) | |  |
| Oropharynx | 38 (16.9) | 273 (17.1) | |  |
| Hypopharynx | 30 (13.3) | 241 (15.1) | |  |
| Larynx | 14 (6.2) | 83 (5.2) | |  |
| **Differentiation, n (%)** | | | 0.497 | |
| Well | 16 (7.1) | 92 (5.7) | |  |
| Moderate | 131 (58.2) | 874 (54.6) | |  |
| Poor | 64 (28.4) | 516 (32.2) | |  |
| Unknown | 14 (6.2) | 119 (7.4) | |  |
| **Tumor stage**^b^**, n (%)** | | | 0.609 | |
| 0/I | 50 (22.2) | 331 (20.7) | |  |
| II | 30 (13.3) | 221 (13.8) | |  |
| III | 34 (15.1) | 200 (12.5) | |  |
| IV | 111 (49.3) | 849 (53.0) | |  |
| **Treatment, n (%)** | | | | |
| Excisional surgery | 161 (71.6) | 1126 (70.3) | | 0.706 |
| Systemic therapy^c^ | 117 (52.0) | 903 (56.4) | | 0.213 |
| Radiation therapy | 115 (51.1) | 866 (54.1) | | 0.401 |
| **Comorbidity** | | | | |
| CAD | 20 (8.9) | 165 (10.3) | | 0.509 |
| COPD | 30 (13.3) | 234 (14.6) | | 0.608 |
| CHF | 7 (3.1) | 49 (3.1) | | 0.967 |
| CVD | 14 (6.2) | 123 (7.7) | | 0.436 |
| CKD | 50 (22.2) | 271 (16.9) | | 0.051 |
| HTN | 75 (33.3) | 544 (34.0) | | 0.848 |
| DM | 49 (21.8) | 367 (22.9) | | 0.701 |
| **Note:**  ^a^ *p* values were calculated by using the chi-square test.  ^b^ Tumor stage was assessed based on the 8th Edition of the American Joint Committee on Cancer staging system  ^c^ Systemic therapy included chemotherapies, cetuximab (an anti-epidermal growth factor receptor antibody) and immune checkpoint inhibitors.  **Abbreviations:**  HBsAg, hepatitis B surface antigen; HNSCC, head and neck squamous cell carcinoma; +, positive; -, negative; CAD, coronary artery disease; COPD, chronic obstructive pulmonary disease; CHF, congestive heart failure; CVD, cardiovascular disease; CKD, chronic kidney disease; HTN, hypertension; DM, diabetes mellitus | | | | |

| **Table 2. Hepatic and virologic characteristics of the HBsAg (+) and HBsAg (-) patients with HNSCC** | | | |
| --- | --- | --- | --- |
|  | **HBsAg (+) (n = 225)** | **HBsAg (-) (n = 1601)** | ***p*** |
| Cirrhosis^c^, n (%) | 26 (11.6) | 58 (3.6) | <0.001^a^ |
| Anti-HCV (+), n (%) | 16 (7.1) | 117 (7.3) | 0.915^a^ |
| ALT (U/L) | 46.6 ± 62.9 | 33.8 ± 37.2 | 0.003^b^ |
| AST (U/L) | 38.5 ± 39.1 | 32.1 ± 38.2 | 0.019^b^ |
| Total Bil. (mg/dL) | 0.7 ± 1.2 | 0.6 ± 0.5 | 0.120^b^ |
| ALKP (U/L) | 111.1± 57.0 | 113.3 ± 59.0 | 0.600^b^ |
| Alb (g/dL) | 4.0 ± 0.7 | 4.0 ± 0.7 | 0.940^b^ |
| INR | 1.05 ± 0.2 | 1.01 ± 0.1 | 0.018^b^ |
| Cr (mg/dL) | 1.04 ± 0.6 | 1.07 ± 0.7 | 0.528^b^ |
| eGFR (mL/min/1.73 m^2^)^d^ | 84.5 ± 26.3 | 81.9 ± 26.3 | 0.178^b^ |
| Initial hepatic dysfunction^e^, n (%) | 24 (10.7) | 97 (6.1) | 0.009^a^ |
| HBV viral load detectable, n (%) | 96 (42.7) |  |  |
| HBV active carrier^f^, n (%) | 45 (20.0) |  |  |
| HBV treatment received, n (%) | 120 (53.3) |  |  |
| Entecavir | 102 (45.3) |  |  |
| Telbivudine | 18 (8.0) |  |  |
| Subsequent hepatic dysfunction, n (%) | 70 (31.1) | 420 (26.2) | 0.122^a^ |
| HBV reactivation^g^ | 14 (6.2) |  |  |
| **Note:**  Numerical data are shown as mean ± standard error of mean.  ^a^ *p* values were calculated by using the chi-square test.  ^b^ *p* values were calculated by using the independent sample t-test  ^c^ Cirrhosis was defined based on typical radiologic findings (e.g., hepatic nodularity, massive ascites and splenomegaly).  ^d^ eGFR was measured with the modification of diet in renal disease equation  ^e^ Hepatic dysfunction was defined as grade 1 or above of hepatic toxicity based on the Common Terminology Criteria for Adverse Events versions 5.0.  ^f^ HBV active carrier was defined as HBV DNA level > 2000 IU/mL.  ^g^ HBV reactivation was diagnosed as (1) a 100-fold increase in viral load compared to the baseline level, (2) DNA level ≥ 1000 IU/mL with undetectable level at baseline, or (3) DNA level ≥ 10,000 IU/mL if the baseline level was unavailable.  **ABBREVIATIONS:** HBsAg, hepatitis B surface antigen; +, positive; -, negative; HNSCC, head and neck squamous cell carcinoma; HCV, hepatitis C virus; ALT, alanine aminotransferase; AST, aspartate aminotransferase; Total Bil., total bilirubin; ALKP, alkaline phosphatase; Alb, albumin; INR, international normalized ratio; Cr, creatinine; HBV, hepatitis B virus; eGFR, estimated glomerular filtration rate | | | |
